# Time reproduction, bisection and doubling: a novel paradigm to investigate the effect of the internal clock on time estimation

**DOI:** 10.1007/s00426-022-01745-0

**Published:** 2022-10-01

**Authors:** Davide Momi, Giulia Prete, Adolfo Di Crosta, Pasquale La Malva, Rocco Palumbo, Irene Ceccato, Emanuela Bartolini, Riccardo Palumbo, Nicola Mammarella, Mirco Fasolo, Alberto Di Domenico

**Affiliations:** 1 Krembil Institute for Neuroinformatics, Toronto, Canada; 2 Department of Psychological, Health and Territorial Sciences, Università degli Studi G. d’Annunzio Chieti-Pescara, Chieti, Italy; 3 Department of Neuroscience, Imaging and Clinical Sciences, Università degli Studi G. d’Annunzio Chieti-Pescara, Chieti, Italy

## Abstract

Time perception is not always veridical, but it can be modulated by changes in internal and external context. The most-acknowledged theory in this regard hypothesises the existence of an internal clock allowing us to subjectively estimate time intervals. The aim of the present study is to investigate the possible effect of such an internal clock, measured as the ability to reproduce a target duration, in the mental manipulation of time: 63 healthy participants were asked to Bisect and to Double reference time intervals, besides Reproducing them. Moreover, to investigate whether time processing might be predicted by individual differences, handedness, anxiety, and personality traits were also assessed by means of standardized questionnaires. Results show that participants correctly Reproduce time intervals (internal clock), but they overestimate time intervals during Bisection and underestimate them during Doubling. We explain this unexpected pattern of results as a kind of aftereffect, due to the short-term retention (adaptation) to the subjective representation of shorter (Bisection) vs longer (Doubling) intervals, respectively. Moreover, hierarchic regression models reveal that some personality traits can predict Bisection accuracy, but they clearly show that the best predictor for both Bisection and Doubling is the accuracy in Reproducing time intervals, confirming the fundamental role of the internal clock in time estimation. We conclude that time estimation is a unique skill, mostly independent from inter-individual differences, and the new paradigms introduced here (bisection vs doubling) reveal that the correct functioning of the internal clock also explains the ability to mentally manipulate the time.

## Introduction

Time perception is an automatic process, but the accuracy in expressing temporal judgment differs among individuals, also in accordance with external environment and internal conditions (Matthews & Meck, 2016). Time scans the moments in our existence, so that the ability to correctly estimate a time interval is crucial for our daily activities (Kononowicz et al., 2018). Given its transversal impact on all our experiences, time perception is one of the topic most investigated in psychology, physiology and neuroscience (Grondin, 2008), nevertheless it still remains controversial. Indeed, conversely to other psychological dimensions, time perception poses a number of unique challenges: for instance—differently from other senses (vision, hearing, smell, etc.)—neither a specific organ nor a single cerebral area have been identified as responsible for time processing (Vroomen & Keetels, 2010). Moreover, varying scales (i.e., from milliseconds to decades) make it really complicated the conceptualization of a single neural substrate or a simple information processing framework of timing (Buonomano, 2007). Nevertheless, different models for time processing have been proposed in the literature. Among these, the Scalar Expectancy Theory, SET (Gibbon et al., 1984a, 1984b), is considered one of the most prominent theoretical accounts of timing. The theory integrates different aspects of human cognition with the psychophysical properties of timing, proposing three interrelated stages of analyses. In particular, the first stage (clock) would be associated with timing: here, an internal pacemaker (counter) would monitor the passage of time. Then, the second stage (memory) would be responsible for a storage of the information just processed, allowing a subjective experience of time. Lastly, the third stage (decision) would be related to the response, thanks to a comparison between the current-objective time and the remembered-subjective time, allowing the selection of an appropriate outcome. Over the past 2 decades, attempts have been made also in identifying brain systems involved in temporal processing, and links have been established between the SET model and neuroanatomical structures undelaying each cognitive stage. The basal ganglia, especially the striatum and pallidum, have been identified as two pivotal hubs of the early raw representation of the temporal interval (Lemoine et al., 2021; Malapani et al., 1998), representing the counter in the SET model (clock stage). Then, the prefrontal cortex would be responsible for the discrimination between internal and external information (Mammarella et al., 2017) and for the raw representation for all the duration of the time interval (Brody et al., 2003), and it would send the processed representation of the interval to the posterior cortex (i.e., inferior parietal lobule and medial temporal lobe; Leon & Shadlen, 2003; Prete et al., 2021), where the final representation of time intervals would be measured, quantified and stored in memory (memory stage). Finally, within the frontoparietal network, the system would compare the new interval with intervals previously stored in memory, and this comparison would drive the behavior (decision stage). This pioneering model has received support also in recent years (Prete et al., 2021) and it has been also integrated, for instance, in a very recent biophysical model (Zemlianova et al., 2022), suggesting the existence of multiple units, allowing the temporal analysis to be translated into the spatial domain, which in turn translates count to a time estimate.

A shared substrate for both temporal and spatial encoding is the basis of another authoritative model proposed by Walsh (Walsh, 2003), aiming to explain the processing of magnitude in general. In fact, Walsh proposed that a common neural substrate exists for time, space and numbers, involving a frontoparietal network. A mole of evidence further validated such model, both at behavioral and cerebral levels, using different paradigms and tasks (Fias, 1996; Macnamara et al., 2018; Prete, 2020; Prete & Tommasi, 2020; Prete et al., 2021), also revealing that duration estimates can vary according to task demands (Droit-Volet et al., 2011). In this model, a left-to-right mental representation is hypothesized, corresponding to a small-to-large distribution of quantities (Dehaene et al., 1993; Walsh, 2003), which has been labeled Mental Number Line (MNL). In the MNL, quantities would be mentally placed from the leftmost to the rightmost portion of a horizontal hypothetical line, in accordance with their smaller/larger quantities, respectively. In this view, independently from the specific content of the magnitude (time, space, and so on), when we are asked to processed quantities, we would automatically place smaller/larger quantities on this MNL, according to their relative weight (from left: smaller, to right: larger). Accordingly, a specific Mental Time Line (MTL) has been proposed, corresponding to the same left-to-right mental representation for small-to-large time intervals, respectively (Droit-Volet & Coull, 2015).

In this framework, the present study was aimed to evaluate whether time representation might be considered a dynamic dimension which might be potentially affected by the environment requests, starting from the hypothesis of a MTL. In particular, we introduced a novel paradigm to test time perception, asking participants either to reproduce, bisect or double a given interval duration (the reference interval). We expected that, in accordance with the left-to-right disposition of quantities (Dehaene et al., 1993; Droit-Volet & Coull, 2015; Walsh, 2003), participants would underestimate the duration when they would be required to bisect the test interval (with smaller quantities mentally placed on the leftmost portion of the MTL), and that they would overestimate the duration when required to double the test interval (with larger quantities mentally placed on the rightmost portion of the MTL), with respect to the duration of the reference stimulus. The reproduction condition (same duration for reference and test interval) was used as a control condition to quantify the objective skill to process time, which would be based on the three stages suggested by the SET model (Gibbon et al., 1984a, 1984b). The paradigm can be considered as a revisitation of the classical paradigms in which participants are asked to categorize a second (test) stimulus as same or different in duration with respect to a first (reference) stimulus (Capizzi et al., 2022; Prete et al., 2021). Furthermore, this comparison task has been already used only in reproduction paradigms (reference and test stimuli had the same duration), and in bisection paradigms (test stimuli lasting a half than the reference one; Kopec & Brody, 2010). In the present study, instead, participants were asked to actively “produce” a specific time interval, which could be the same (reproduction), a half (bisection) or twice (doubling) in duration with respect to a reference interval. A correlation analysis is also carried out among the three tasks (reproduction, bisection and doubling) to verify the possibility of an overall ability in mental time manipulation: we expected that a better performance in reproducing time intervals can correlate with a better performance in manipulating the same intervals (bisection and doubling). Furthermore, besides introducing the doubling condition and the active adjustment task, we also measured handedness, anxiety and personality traits in a sample of healthy participants. In fact, a possible influence of handedness on the processing of magnitudes has been suggested (Serrien & Spapé, 2022), including time (Hancock, 2011), and a very recent study based on the SET model revealed that, for instance, in older adults, the accuracy in time scanning would be compromised due to a slower clock (stage 1 of the SET model), accumulating less pulses, and leading to a slower scanning of time intervals (stages 2 and 3; Capizzi et al., 2022; Droit-Volet et al., 2019). Moving from these premises, we wondered whether more anxious participants have a faster clock, resulting in more pulses accumulated and thus in a faster subjective scanning of time: we expected anxiety as a predictor of the accuracy in timing paradigms, hypothesizing a wider underestimation of intervals in participants with higher anxiety scores, with faster counter resulting in anticipatory responses. Finally, starting from some evidence of a different time processing in accordance with some personality traits (in particular: neuroticism, Witowska et al., 2020; and extraversion, Bisson & Grondin, 2020; Rammsayer, 1997), we also administered a personality questionnaire to verify whether specific personality traits could be associated with a better performance in time processing. It has been found that higher levels in psychoticism and emotional instability (neuroticism) are related to higher overestimation of time intervals (Kirkcaldy, 1984), and that higher levels of extroversion lead to a greater error rate in time judgments, so that it has been proposed that extravert individuals generate an active inhibition processing more quickly, and that they also switch-off this inhibition more slowly, than introvert persons (Eysenck, 1959). Starting from these findings, we hypothesized larger errors in time estimation for participants with higher levels of neuroticism and extraversion, and—conversely—a better performance in participants with higher scores in conscientiousness, due to a higher control of the internal clock.

## Materials and methods

### Participants

The study was carried out by 63 university students (34 females), with a mean age of 21.09 years (± 2.53) and a mean scholarity of 14.17 years (± 2.25). All participants were recruited at the University of Chieti and they took part in the study as volunteers. They signed an informed consent prior to take part in the study, which was approved by the local ethical committee. All participants self-reported normal or corrected to normal vision and absence of auditory impairments, neurological and/or psychiatric conditions.

### General procedure and psychological assessment

Each participant carried out two sessions: the first session was carried out online and it consisted in the self-administration of three questionnaires; the second session was carried out in the laboratory, and it consisted in the experimental task.

The online assessment (session 1) required approximately 20 min to be completed and it was carried out independently by each participant, at home. The psychological assessment was performed by using Qualtrics XM (Qualtrics Labs, Inc.; www.qualtrics.com), a subscription software for collecting and analyzing data (Snow & Mann, 2013): once recruited, each participant received an email with a link to the online items. Another statement in the email explained that participation in the study was voluntary and that the responses given in this session could not be wrong, but they measured personal attitudes. A final statement informed that the battery was completed and that the responses will be automatically recorded and sent to the server. Thus, an experimenter remotely monitored the amount of time participants spent filling out each questionnaire and that all the responses were correctly filled in.

The first test was administered to measure the laterality bias of the sample (Edinburgh Handedness Inventory; Salmaso & Longoni, 1983), starting from the evidence of a possible influence of handedness on the processing of magnitudes (Serrien & Spapé, 2022), including time (Hancock, 2011). The test consists of 13 items describing different motor activities and participants are asked to specify if the described activity is preferentially or absolutely carried out using the left or the right hand. The final score ranges from − 100 to + 100 (0 representing an absence of laterality preference), with higher scores indicating a more pronounced preference for the right hand. Means and standard deviations for all the test administered online in session 1 are reported in Table 1.

**Table 1:** Mean and standard deviation of the sample in the assessment of handedness (Edinburgh Handedness Inventory), anxiety (State-Trait Anxiety Inventory) and personality traits (Big Five Questionnaire)

Scale	Subscale	Mean	Standard deviation
Edinburgh Handedness Inventory		54.79	33.68
State-Trait Anxiety Inventory	State anxiety	44.02	12.05
	Trait anxiety	49.66	13.92
Big Five Questionnaire	Openness to experience	49.48	10.11
	Conscientiousness	52.79	11.49
	Extraversion	48.84	10.68
	Agreeableness	47.78	10.98
	Neuroticism	47.54	10.44

Anxiety was measured by means of the State-Trait Anxiety Inventory (Lazzari & Pancheri, 1980): the STAI consists of 40 items, 20 for assessing participants’ feeling at the present moment (state anxiety) and 20 for evaluating their frequent state (trait anxiety). All items are rated on a 4-point Likert scale (from “almost never” to “almost always”), with higher scores indicating greater anxiety.

Finally, personality traits were assessed by using the Big Five Questionnaire (Caprara et al., 1993), measuring personality traits as defined by the Five Factor Theory of Personality, by means of 132 items rated using a 5-point Likert scale, from 1 (very false for me) to 5 (very true for me). Specifically, the BFQ measured five different scales, corresponding to five personality traits: 1) Openness to experience (inventive/curious vs consistent/cautious), 2) Conscientiousness (efficient/organized vs extravagant/careless), Extraversion (outgoing/energetic vs solitary/reserved), Agreeableness (friendly/compassionate vs critical/rational), Neuroticism (sensitive/nervous vs resilient/confident).

The experimental task was carried out in the laboratory (session 2): once completed the first session, each participant was invited to carry out a second session in which they had to reproduce time intervals in a computerized task. The experimental session lasted about 10 min.

### Stimuli and procedure

**Fig. 1:**
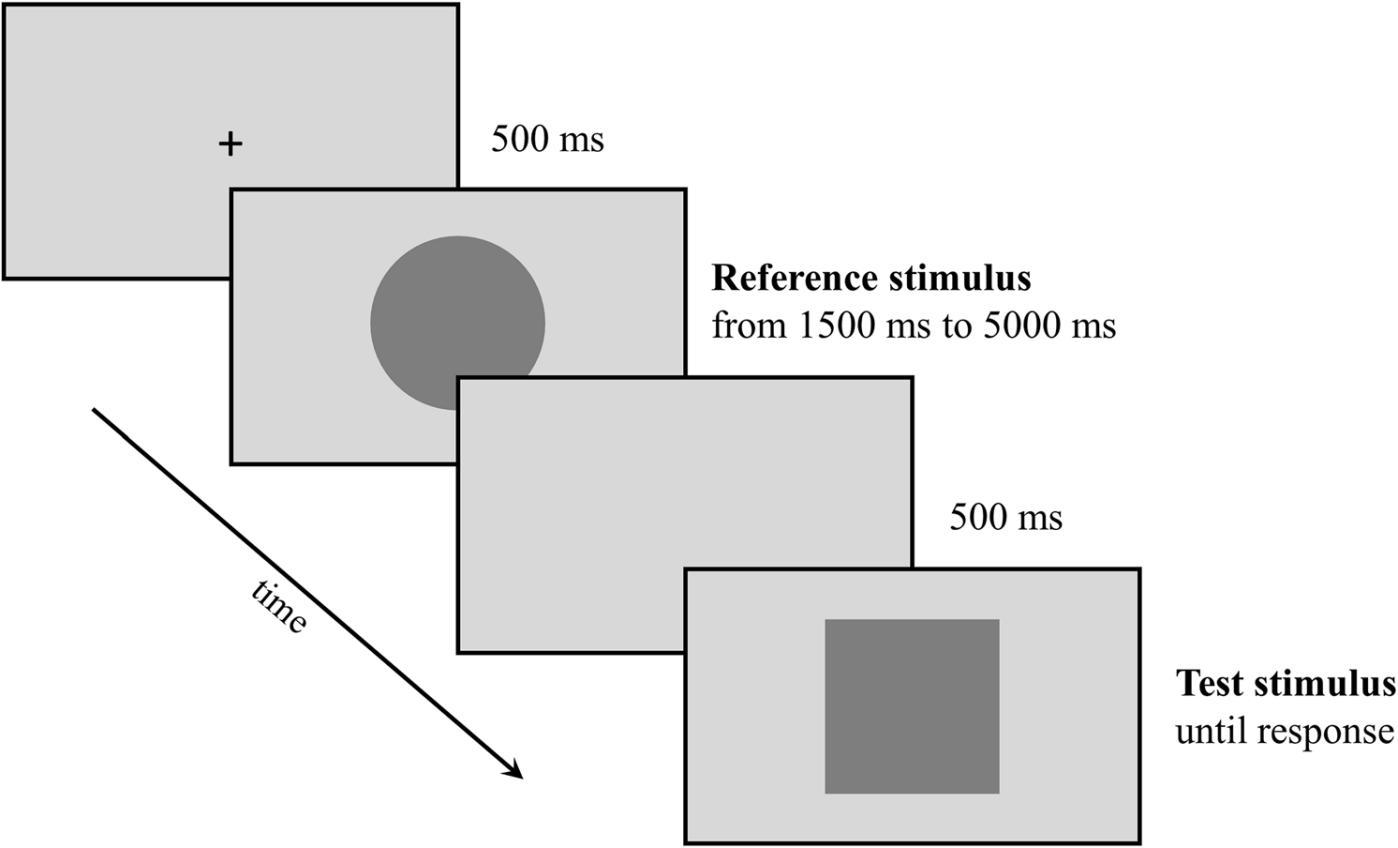
Schematic representation of the experimental procedure. During the task, participants were presented with a reference stimulus which duration was randomized between 1500 and 5000 ms (step: 500 ms), and were asked to press the spacebar when the test stimulus duration reached either the same duration of the reference stimulus (Reproduction condition), half of the duration of the reference stimulus (Bisection condition), or twice the duration of the reference stimulus (Doubling condition)

The experimental task was administered by using E-prime 2.0 software (Psychology Software Tools Inc.; www.pstnet.com/eprime) on a Windows laptop PC. Each trial started with a fixation cross presented in the center of the screen for 500 ms, followed by a gray circle (the reference stimulus), which duration varied in a range from 1500 to 5000 ms, with steps of 500 ms (each of the eight durations was presented 15 times in a random order). Then, after 500 ms of delay, a gray square appeared and the participant was required to respond by pressing the spacebar to indicate the duration of the square, accordingly with the block condition (see Fig. 1). Specifically, the task consists of three separate block conditions in a within-subjects design (all participants carried out each of the three blocks): Bisection, Reproduction, and Doubling.

Depending on condition, participants were asked to press the spacebar when the second stimulus duration reached the half (Bisection), the same (Reproduction) or twice (Doubling) the reference stimulus duration. A set of 120 trials was administered, including 40 trials for each block, and both blocks order, reference duration and stimulus type (square or circle as reference) were randomized across participants.

## Results

### Statistical analyses

Statistical analyses were carried out using SPSS software version 20 (IBM Corp, 2011). The dependent variable was the error in time estimation, namely the difference between the perceived duration expressed by the participant and the real duration of the reference stimulus. This difference was normalized by computing a T-corrected score (Mc Conchie & Rutschmann, 1970; Treisman, 1963) with the following formula:

$$\mathrm{T}-\mathrm{corrected}=\frac{\mathrm{T\,estimated}-\mathrm{ T\,standard }}{\mathrm{T\,standard}}$$

where T estimated is the mean duration estimate provided by a participant for a given reference stimulus duration (T standard). This normalization provided information about both the extent and the direction of the error of temporal estimation for each task (Bisection, Reproduction and Doubling) regardless of the interval duration. Negative and positive values indicate that the test stimulus had been reproduced either shorter (underestimation) or longer (overestimation) than the real duration, respectively.

### Accuracy in time estimation

**Fig. 2:**
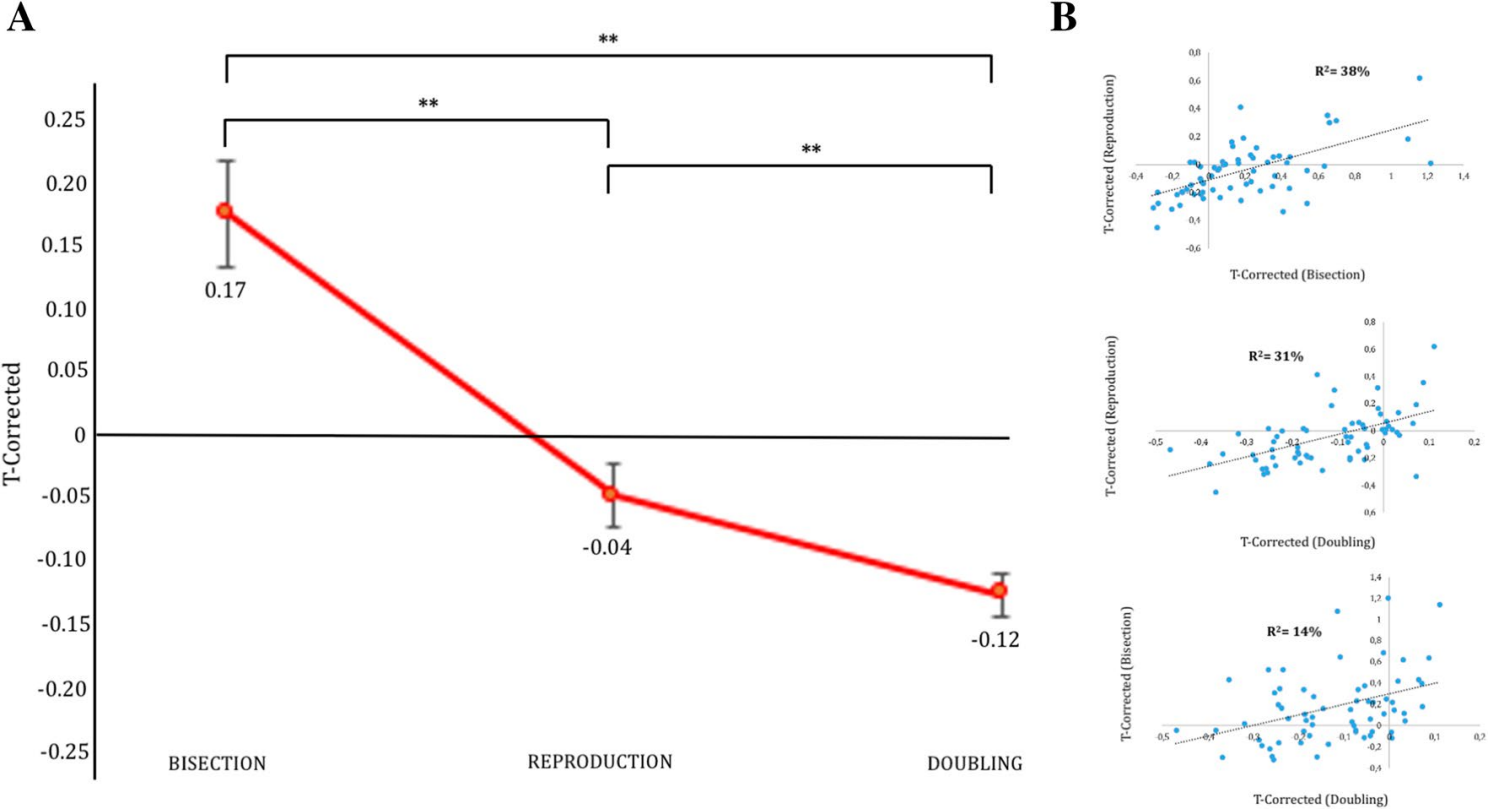
T-corrected measured in each condition. (A) The Tc scores display a significant overestimation for Bisection and a significant underestimation for Doubling (with Reproduction not differing from 0). (B) Scatter plots show significant correlations between: Reproduction and Bisection (upper panel), Reproduction and Doubling (central panel), Bisection and Doubling (bottom panel)

As a first step, three single-sample t-tests were carried out, comparing the T-corrected value (Tc) for each condition with the correct performance (Tc = 0; see Fig. 2A). Results revealed that Tc did not differ from 0 in the Reproduction condition (Mean ± Standard Error: – 0.046 ± 0.02; *t*_(62)_ = – 1.89). Tc was instead significantly higher than 0 in the Bisection condition (0.176 ± 0.04; *t*_(62)_ = 4.21, *p* < 0.001) and it was lower than 0 in the Doubling condition (– 0.126 ± 0.02; *t*_(62)_ = – 7.58, *p* < 0.001).

Then, an Analysis of Variance (ANOVA) was carried out, using Condition (Bisection, Reproduction, Doubling) as a within-subjects factor and Tc as the dependent variable. As shown in Fig. 2A, the significant main effect of Condition (*F*_(2, 124)_ = 49.07, *η*_*p*_^*2*^ = 0.44, *p* < 0.001) revealed that participants’ estimation was larger in the Bisection condition than in both the Reproduction and the Doubling conditions (*p* < 0.001 for both comparisons), and that it was smaller in the Doubling than in the Reproduction condition (*p* = 0.033).

Finally, Pearson correlations were performed to test the relationship between individual performance across the different conditions (Bisection, Reproduction, Doubling; Fig. 2B): results showed that the performance in the three conditions were correlated to each other (Reproduction vs Bisection: *R*^*2*^ = 0.37, *p* < 0.001; Reproduction vs Doubling: *R*^*2*^ = 0.31, *p* < 0.001; Bisection vs Doubling: *R*^*2*^ = 0.16, *p* = 0.001).

### Individual differences in mental manipulation of time

The last step of the analysis was aimed to investigate whether individual variables can predict the performance in bisecting and doubling time intervals. To this aim, hierarchical multiple regressions was performed, with Tc (errors) for Bisection and Doubling included in two distinct models, each with five blocks of variables. In particular, demographic variables (age and scholarity) were included in step 1 as controls; handedness was included in step 2 to verify the hypothesis of the effect of the MTL (which seems to be dependent upon laterality preference) on the performance measured; then, trait and state anxiety were included in step 3 to verify the possibility of an acceleration/reduction of the internal clock as a result of individual anxiety; the five personality traits measured (Openness to experience, Conscientiousness, Extraversion, Agreeableness, Neuroticism) were considered in step 4 to control for the effect of personal attitude; finally, errors in Reproduction (Tc) was included in step 5 to verify whether, after having controlled the psychological effects, the specific timing ability is responsible for the performance in tasks requiring a mental manipulation of time intervals.

Concerning Model 1 (Bisection; Table 2), results showed that the first three steps did not explain a significant amount of variance in Bisection performance. When personality traits were entered in the model (Step 4), the amount of explained variance raised to 28%. The coefficients inspection revealed that only Conscientiousness and Extraversion positively predicted the outcome, whereas Openness to experience negatively predicted the outcome. However, when the performance in Reproduction was entered in the model (Step 5), only Extraversion and Openness to experience remained statistically significant. The variable entered in Step 5 contributed to explain 17% of the variance, with reproduction error positively predicting bisecting error.

**Table 2:** Summary of regression analysis for variables predicting Tc (error) in the Bisection condition (Model 1)

Variable	Bisection																			
	Step 1				Step 2				Step 3				Step 4				Step 5			
	*B*	*SE*	*β*	*t*	*B*	*SE*	*β*	*t*	*B*	*SE*	*β*	*t*	*B*	*SE*	*β*	*t*	*B*	*SE*	*β*	*t*
Scholarity	0.000	0.022	− 0.003	− 0.018	− 0.002	0.023	− 0.014	− 0.089	− 0.016	0.023	− 0.109	− 0.701	0.005	0.023	0.031	0.196	− 0.007	0.02	− 0.047	− 0.342
Age	− 0.002	0.02	− 0.015	− 0.097	− 0.002	0.02	− 0.018	− 0.121	0.003	0.02	0.022	0.145	− 0.007	0.018	− 0.05	− 0.358	− 0.006	0.016	− 0.043	− 0.358
Handedness					− 0.001	0.001	− 0.076	− 0.576	− 0.001	0.001	− 0.084	− 0.652	− 0.002	0.001	− 0.163	− 1.364	− 0.001	0.001	− 0.152	− 1.472
Trait anxiety									− 0.007	0.003	− 0.276	− 2.02	− 0.005	0.003	− 0.218	− 1.799	− 0.002	0.003	− 0.098	− 0.899
State anxiety									− 0.002	0.004	− 0.074	− 0.546	0.000	0.005	− 0.014	− 0.084	0.000	0.004	0.008	0.054
Extraversion													0.01	0.004	0.319	2.265*	0.009	0.004	0.287	2.343*
Agreeableness													− 0.005	0.004	− 0.16	− 1.149	− 0.002	0.004	− 0.054	− 0.437
Conscientiousness													0.009	0.004	0.322	2.307*	0.005	0.004	0.172	1.367
Neuroticism													0.000	0.005	0.013	0.083	− 0.001	0.004	− 0.029	− 0.213
Openness to experience													− 0.014	0.005	− 0.437	− 2.654*	− 0.011	0.005	− 0.331	− 2.285*
Tc Reproduction																	0.803	0.187	0.473	4.287**
*Adjusted R*^*2*^	− 0.033				− 0.045				0.011				0.254				0.441			
*R*^*2*^* change*	0.000				0.006				0.085				0.284				0.166			
*F for change in R*^*2*^	0.008				0.332				2.655				4.721*				18.379**			

*p < 0.05

**p < 0.001

Concerning Model 2 (Doubling; Table 3), results showed that the first four models were not statistically significant, indicating that none of the variables entered predicted Doubling performance. When performance in Reproduction was entered in the model (Step 5), it explained a significant 23% of the variance in the outcome and coefficients inspection showed that Reproduction error positively predicted Doubling error.

**Table 3:** Summary of regression analysis for variables predicting Tc (error) in the Doubling condition (Model 2)

Variable	Doubling																			
	Step 1				Step 2				Step 3				Step 4				Step 5			
	*B*	*SE*	*β*	*t*	*B*	*SE*	*β*	*t*	*B*	*SE*	*β*	*t*	*B*	*SE*	*β*	*t*	*B*	*SE*	*β*	*t*
Scholarity	0.004	0.009	0.06	0.405	0.003	0.009	0.054	0.356	0.001	0.009	0.025	0.160	0.004	0.01	.065	0.37	− 0.002	0.009	− 0.026	− 0.171
Age	− 0.011	0.008	− 0.214	− 1.439	− 0.011	0.008	− 0.216	− 1.442	− 0.008	0.008	− 0.146	− 0.97	− 0.008	0.008	− 0.146	− 0.921	− 0.007	0.007	− 0.138	− 1.024
Handedness					0.000	0.001	− 0.045	− 0.346	0.000	0.001	− 0.076	− 0.589	− 0.001	0.001	− 0.143	− 1.057	− 0.001	0.000	− 0.13	− 1.132
Trait anxiety									0.000	0.001	− 0.022	− 0.16	0.000	0.001	0.021	0.15	0.002	0.001	0.163	1.348
State anxiety									− 0.003	0.001	− 0.258	− 1.917	− 0.003	0.002	− 0.257	− 1.338	− 0.003	0.002	− 0.231	− 1.412
Extraversion													0.000	0.002	− 0.032	− 0.198	− 0.001	0.002	− 0.08	− 0.516
Agreeableness													− 0.002	0.002	− 0.179	− 1.134	− 0.001	0.002	− 0.053	− 0.388
Conscientiousness													0.004	0.002	0.323	2.044*	0.002	0.002	0.146	1.039
Neuroticism													− 0.001	0.002	− 0.083	− 0.472	− 0.002	0.002	− 0.133	− 0.881
Openness to experience													− 0.001	0.002	− 0.08	− 0.431	0.001	0.002	0.045	0.282
Tc Reproduction																	0.377	0.002	0.559	4.553**
*Adjusted R*^*2*^	0.004				− 0.011				0.024				0.043				0.306			
*R*^*2*^* change*	0.036				0.002				0.065				0.094				0.232			
*F for change in R*^*2*^	1.117				0.119				2.07				1.22				20.73**			

*p < 0.05

**p < 0.001

## Discussion

Time processing is a complex ability, susceptible of inter-individual differences: subjective experience of time can differ from objective time, and it can also differ among persons. In the present study, we investigated not only the ability to reproduce, after a brief delay, the time interval just experienced, but also the ability to mentally manipulate this time interval, by asking participants to reproduce it as either lasting a half of time or twice with respect to the reference interval. The first result of the present study is that participants correctly reproduced a short time interval (from 1500 to 5000 ms), confirming a kind of internal clock which keeps track of time. Positive correlations among the three conditions (reproduction, bisection and doubling), moreover, confirm an overall ability in mentally manipulating time intervals. In accordance with the SET model (Gibbon et al., 1984a, 1984b), we can conclude that in this condition all the three stages included in the model correctly run: the internal counter (clock) monitor the passage of time (stage 1), it allows a storage of the information just processed into the memory (stage 2), and after a comparison between the current-objective time and the remembered-subjective time, the appropriate response is selected and produced (step 3).

Second, the present results also showed that such a model is valid only in the Reproduction condition, when the interval just experienced has to be reproposed with its exact duration. However, when participants were asked to mentally manipulate the interval duration, they overestimated time intervals when they should mentally bisect the durations, but they underestimated them when they should mentally double the durations. This pattern of results revealed not only that the internal clock can be useful when time has to be linearly scanned, but also that it can fail when time has to be mentally manipulated, furthermore it appears to be in contrast with a Mental Time Line (Droit-Volet & Coull, 2015). According to the MTL, in fact, we expected a shift on the left side of the hypothetical mental line during bisection (when the time interval must be mentally divided by two), leading to an underestimation, and a shift on the right side of the MTL during doubling (when the time interval must be mentally multiplicated for two), with a consequent overestimation. We speculate that this pattern can be due to the fact that a left-to-right mental disposition of time (MTL) occurs mainly in perceptual tasks, whereas in the present task, in which a short-term retention is required, information stored in memory is not subjected to this kind of mental spatialization. We hypothesized, instead, that the convergence of the performance toward a middle interval (i.e., underestimation during bisection and overestimation during doubling) could be viewed as a kind of aftereffect. In this perspective, when required to reproduce an interval longer than that just presented (doubling condition), participants tend to mentally shorten the estimated duration, whereas when required to reproduce an interval shorter than that just presented (bisection condition), they tend to lengthen the estimated duration. A similar pattern has been widely documented when participants are asked to compare two intervals and to decide whether the second is shorter/longer than the first one (e.g., Heron et al., 2012; Li et al., 2017): in this condition, it has been found that after being adapted to a long/short reference stimulus, the following test stimulus is judged as shorter/longer than the first one, respectively (Li et al., 2017; Prete et al., 2021). The present results can be viewed in this framework: starting from the correct performance during the Reproduction condition, showing that participants are able in internally scanning the time, we can speculate that in the Bisection condition, participants mentally transformed the perceived reference duration into a shorter duration (half of the reference), conversely in the Doubling condition they mentally transformed the perceived reference duration into a longer duration (twice the reference). Thus, we propose that the short-term retention in memory of these transformed durations (until the response is given, stage 2 of the SET model) acts as a kind of imagined adaptor, leading to the same kind of aftereffect described in perceptual tasks.

Finally, the present results showed that neither age nor scholarity affect the performance, even if it has to be highlighted that the sample tested in the present study is highly homogeneous in both of these variables, and the same is true for handedness, with only 4 left-handed participants. Moreover, no effect of anxiety scores emerged from the present results, confirming previous evidence (Kelly, 2000). However, some personality traits explained the performance in the Bisection condition. It is interesting in this regard to highlight that also the specific effect of personality traits found here largely confirmed previous evidence: for instance, a study involving male participants revealed that, when required to reproduce target intervals, extraverts tended to overestimate time and to make less accurate time judgments when compared to introverts, whereas participants with higher psychoticism scores were less prone to overestimate time intervals and showed better accuracy of temporal reproduction than those with lower psychoticism scores (Rammsayer, 1997). The present results confirm a significant effect of personality traits, at least in time bisection, confirming the central role of extraversion and openness to experience on time processing (see also (Bisson & Grondin, 2020).

Finally, the best predictor for both Bisection and Doubling is anyway the performance in the Reproduction condition. This evidence further supports the abovementioned idea of a functioning clocker, which is at the basis for the correct performance in the Reproduction condition, and agrees with the idea that it is not time scanning the issue leading to a wrong performance in the other two conditions: a person who performs well in the Reproduction has high timing skill. The issue at the basis of the incorrect performance during the Bisection and the Doubling would be an adaptation to the correctly imagined intervals, which have been either bisected or doubled, which in turn leads to the overestimation or underestimation of the test interval. This idea is also in line with the positive correlations found among the three tasks, revealing that participants with a good performance in time reproduction have a good performance in both time bisection and doubling. Further studies are needed to verify such a conclusion, also due to the fact that the active reproduction of a perceived time interval is a poorly exploited paradigm, since the most exploited task in this domain is the passive judgment of the length of a test stimulus as shorter/longer with respect to a reference. Furthermore, even if the bisection procedure has been already used in previous studies, to our knowledge, no previous tasks have been carried out in which participants are asked to actively reproduce a test stimulus lasting twice than the reference. For all these reasons, the present results must be considered an interesting starting point for further explorations of this still debated framework, which is so crucial in our everyday life.

In the context of time perception, several studies have been shown how mental representation of a given interval might be modulated by multiple factors such as mood (Fayolle et al., 2014) and cognitive load (Block & Gruber, 2014). These studies described time perception as a dynamic dimension which might be sensitive to both environment request and specific situation. Moreover, over the last 10 years, psychologists have become increasingly interested in what we might call “time illusions”, which consists in a misrepresentations or manipulations of some temporal aspect of a situation (Jaffe, 2018). In our study, even though participants were able to replicate the time interval accurately, a significant overestimation and underestimation was present when they were asked to bisect or double the time duration. This progressive shift of the internal clock might suggest that, once created, the mental representation of a given interval would be affected by either a bisection or a doubling task where the participants are asked to manipulate the time efference copy to efficiently respond to environment requests. Interestingly, the error was strongly related to the direction of the time projection with participants that either under or overestimate, depending on whether they were asked to bisect or double the time interval. Previous studies reported this effect of direction for other cognitive domains, such as space perception (Bradshaw et al., 1987), which shares neural substrates with time representation (Walsh, 2003). Indeed, numerous findings have shown how, depending on conditions, our brain fails to correspond the time’s true nature (Treisman, 1999). Internal and external factors affect the processing of the metaphysical representation of a given interval and it seems that the brain would be able to counterbalanced accordingly with the situation (Yarrow et al., 2001). Such compensation can lead us into error which might assume different direction (under/overestimation) depending on environment request.

This study showed that brief time intervals can be correctly reproduced after a short delay, providing a further support for an internal clock keeping track of time. The mental manipulation of time—required to bisect and to double the same time intervals—is possibly based on the same cognitive mechanism, as suggested by the positive correlations among the performance collected in each of these tasks, but it does not exactly correspond to the same internal clock. In the present study, in fact, participants overestimated and underestimated time when required to bisect and to double the intervals, respectively. This evidence suggests an adaptation mechanism, according to which the mental representation of time acts as an imagined adaptor, influencing the following response. Finally, the present results also revealed that neither age/scholarity nor anxiety affect time processing, but they showed a role of some personality traits (i.e., extraversion and openness to experience) on time bisection. This last evidence needs to be further investigated in the attempt to shed light on the specific mechanisms underlying this peculiar task. It is surprising in fact to note that personality traits do not affect either time reproduction or doubling, possibly suggesting specific mechanisms—and leading to also hypothesize specific cerebral circuits—involved in this task. This speculation must be specifically investigated both at a cognitive and at a cerebral level, but the results found here suggest this possibility. As specified above, caution is needed concerning the null effects of the demographic data on time processing due to the high homogeneity of the sample tested. In particular, it would be interesting to investigate how time processing change during lifespan, the possible difference between women and men in time manipulation, as well as the possible effect of field of study (scientific vs. humanistic) on time scanning, and following studies should involve participants with different background and demographic features to disentangle the role of such individual features on a domain which is so crucial in our daily life such as time processing.

## Data Availability

The datasets generated and analyzed during the current study are available from the corresponding author on reasonable request.
